# A scoping review of the evaluation and effectiveness of technical assistance

**DOI:** 10.1186/s43058-022-00314-1

**Published:** 2022-06-28

**Authors:** Victoria C. Scott, Zara Jillani, Adele Malpert, Jenny Kolodny-Goetz, Abraham Wandersman

**Affiliations:** 1 Department of Psychological Science, University of North Carolina at Charlotte, 9201 University City Boulevard, Charlotte, NC 28223 USA; 2 University of North Carolina at Charlotte, 9201 University City Boulevard, Charlotte, NC 28223 USA; 3 Wandersman Center, Columbia, USA

**Keywords:** Technical assistance, TA, Technical assistance evaluation, Technical assistance effectiveness, Capacity building, Scoping review

## Abstract

**Background:**

Although the benefits of evidence-based practices (EBPs) for advancing community outcomes are well-recognized, challenges with the uptake of EBPs are considerable. Technical assistance (TA) is a core capacity building strategy that has been widely used to support EBP implementation and other community development and improvement efforts. Yet despite growing reliance on TA, no reviews have systematically examined the evaluation of TA across varying implementation contexts and capacity building aims. This study draws on two decades of peer-reviewed publications to summarize the evidence on the evaluation and effectiveness of TA.

**Methods:**

Guided by Arksey and O’Malley’s six-stage methodological framework, we used a scoping review methodology to map research on TA evaluation. We included peer-reviewed articles published in English between 2000 and 2020. Our search involved five databases: Business Source Complete, Cumulative Index to Nursing and Allied Health Literature (CINAHL), Education Resources Information Center (ERIC), PsycInfo, and PubMed.

**Results:**

A total of 125 evaluation research studies met the study criteria. Findings indicate that publications have increased over the last two decades, signaling a growth in the recognition and reporting of TA. Technical assistance is being implemented across diverse settings, often serving socially vulnerable and under-resourced populations. Most evaluation research studies involved summative evaluations, with TA outcomes mostly reported at the organizational level. Only 5% of the studies examined sustainability of TA outcomes. This review also demonstrates that there is a lack of consistent standards regarding the definition of TA and the level of reporting across relevant TA evaluation categories (e.g., cadence of contact, and directionality).

**Conclusions:**

Advances in the science and practice of TA hinge on understanding what aspects of TA are effective and when, how, and for whom these aspects of TA are effective. Addressing these core questions requires (i) a standard definition for TA; (ii) more robust and rigorous evaluation research designs that involve comparison groups and assessment of direct, indirect, and longitudinal outcomes; (iii) increased use of reliable and objective TA measures; and (iv) development of reporting standards. We view this scoping review as a foundation for improving the state of the science and practice of evaluating TA.

**Supplementary Information:**

The online version contains supplementary material available at 10.1186/s43058-022-00314-1.

Contributions to the literature

- This scoping review draws on two decades of peer-reviewed publications to summarize the evidence on the evaluation and effectiveness of TA.
- The synthesis illuminates four aspects core to enhancing the evaluation of TA: (i) establish a standard definition of TA, (ii) apply robust and rigorous evaluation research designs, (iii) use psychometrically tested and objective measures, and (iv) develop reporting standards.
- Insights from this review provide important knowledge for implementation science frameworks, particularly the *Interactive Systems Framework for Dissemination and Implementation* and the *Evidence-Based System for Innovation Support (EBSIS*).

## Introduction

Although the benefits of evidence-based practices (EBPs) for advancing community outcomes are well-recognized, there are considerable challenges to the use of EBPs in practice, including inaccessible EBP research and publications, resource scarcity, inadequate organizational or leadership support, and limited staff capacity or motivation to engage in EBP efforts [1–5]. Consequently, many EBPs are poorly disseminated, implemented, and sustained across organizational and community settings [2, 6–9]. Recent efforts to reduce barriers to EBPs highlight the critical role of active, collaborative approaches in supporting EBP dissemination and implementation efforts. Technical assistance (TA) is one such approach used worldwide in both public and private sectors [10–12].

Technical assistance refers to an individualized, hands-on approach to capacity building in organizations and communities [13, 14]. This approach involves the provision of tailored guidance by a TA specialist to meet the specific needs of a site(s) through collaborative communication between the TA provider and site(s) or TA recipient(s) [15]. TA services often include a combination of activities such as coaching, consulting, modeling, facilitation, professional development, site visits, and referral to informational resources [16, 17]. The delivery format can vary along multiple dimensions: individualized–group, onsite–virtual, active (high intensity)–passive (low intensity), and peer-to-peer–directed [17]. In addition to supporting the implementation or improvement of an innovation, such as an EBP program, practice, or policy, TA can enhance overall system capacities by empowering staff and improving general organizational or systems processes [13, 18, 19]*.* As a predominant approach to organizational and community improvement, it is also a global strategy for addressing larger-scale, longstanding, and emerging social issues [20], particularly in child welfare, youth development, education, and community health improvement.

Despite its widespread use, identifying and measuring the impacts of TA is challenging due to a lack of consensus regarding the essential features of TA, inherent variability of tailored services, and minimal use of a framework to systematically plan, implement, and evaluate TA [13, 16, 17, 21]. Variations in setting and population characteristics and differences in recipient organizational goals further complicate measuring TA outcomes. Evaluation studies on the impact of TA are sparse relative to the prevalence of TA use, and findings on the effects of TA on program and system-level outcomes are mixed [22].

While previous reviews have examined important links between TA practices and setting outcomes, they are often limited to a particular domain (e.g., global health [21]) or implementation goal (e.g., uptake of EBP [23]). West and colleagues reviewed the scientific literature on evaluations of TA between 2000 and 2010 to examine its effectiveness in furthering global health [21]. Based on a synthesis of 23 articles, they reported an increasing number of scholarly evaluations of TA but limited evidence of TA effectiveness. The review identified challenges associated with TA provision related to cost effectiveness, managing the growing amount of scientific and technical knowledge, and sustaining global TA supports. The authors concluded that evaluating the quality, process, cost-effectiveness, and impact of TA is an integral component of TA and encouraged more rigorous evaluations of TA efforts. Dunst and colleagues [23] conducted a quantitative analysis to examine the effects of TA on adopting evidence-based and evidence-informed practices. Inclusive of 25 studies and evaluations, their review focused on relating 25 core TA elements (e.g., decision-making, TA resources, and provider feedback) to evaluation outcomes (e.g., adoption and use of targeted practice). They only included TA literature with between-groups or between-condition comparisons to permit effect size calculations. Broadly, results showed that a subset of core TA elements was related to between-group and between-condition differences in effect sizes for TA outcomes. More intensive TA had more robust effects on targeted outcomes compared to less intensive TA. Evaluations that monitored fidelity of both TA practices and intervention practices had larger effect sizes than those that were less attentive to those two core elements.

Though prior studies have contributed valuable insights to examining the field of TA, to our knowledge, no review has comprehensively examined outcomes of TA across varying implementation contexts and capacity building aims. Further, no reviews have systematically synthesized how evaluators conduct evaluations of TA (e.g., formative versus process versus summative evaluation). The increasing number of TA evaluation studies calls for scoping reviews that summarize TA practices and knowledge as well as illuminate trends.

The aims of our scoping review are to (i) document the methodology of evaluation research about TA and (ii) summarize findings associated with TA. Through this review, we seek to identify practical opportunities for improving the implementation, evaluation, and study of TA. Additionally, this scoping review provides important concepts and evidence for furthering capacity building in implementation science frameworks. For example, TA is a key mechanism in the *Interactive Systems Framework for Dissemination and Implementation*, which reflects the role of a support system in building the capacity of the delivery system [24]. TA is also a core element in the *Evidence-Based System for Innovation Support* (EBSIS) framework, which emphasizes the need for support to be evidence-based to effectively achieve targeted implementation outcomes [10]. We view the current study as a foundation to improving the state of the science and practice of evaluating TA.

## Methods

We use a scoping review methodology to map existing research on TA evaluation. A scoping review is designed to identify knowledge gaps, describe the body of literature, clarify concepts, or investigate research conduct [25]. Like a systematic review, a scoping review involves a structured, predefined process that is systematic, transparent, and reproducible, which includes steps to reduce error and increase the reliability of findings [25].

Our review was guided by Arksey and O’Malley’s [26] methodological framework, which includes six stages: (1) identifying the research question; (2) identifying relevant studies; (3) selecting studies; (4) charting the data; (5) collating, summarizing, and reporting results; and (6) consulting with relevant stakeholders. Additionally, we incorporated suggested methodological enhancements to the six-stage framework (e.g., using an iterative team-based approach to select studies and extract data, incorporating a quantitative and qualitative summary of data, and employing consultation throughout the review process [27, 28]). The study protocol is available via the corresponding author.

### Stage 1: Identifying the research question

The development of our research questions began with a collaborative dialog among our research team members, who are TA providers and researchers with expertise in TA and implementation science. We used an iterative process to formulate and refine research questions based on research literature and practice-based experience. We identified the following research questions:

*Research question 1 (RQ1)*: How has TA been evaluated in the scientific literature? (1a and 1b)

RQ1a: What measurement approaches have been used to assess TA?

RQ1b: How have TA outputs and outcomes been conceptualized, and what are notable trends?

*Research question 2 (RQ2)*: To what extent has TA provision resulted in sustainable improvements in organizations and communities?

### Stage 2: Identifying relevant studies

#### Databases and search strategy

The research team generated an initial set of keyword searches based on the research questions and the research team’s collective experience with TA literature. We piloted the initial set of keywords using two databases, PubMed and PsycInfo. This pilot search was limited to (i) English-only articles (the fluent language of the researchers), (ii) publication time frame (January 2000 to June 2020), and (iii) peer-reviewed articles. We examined titles, abstracts, and index terminology to refine the search terms and ensure that we captured relevant literature for review. This process produced the final search terms: “technical assistance” AND “assessment” OR “effectiveness” OR “evaluat*” OR “impact” OR “measurement” OR “outcome*” OR “output*” OR “questionnaire” OR “result*” OR “scale” OR “tool.” Then, we entered the final search terms into three additional databases relevant to the evaluation of TA: Education Resources Information Center (ERIC), Business Source Complete, and Cumulative Index to Nursing and Allied Health Literature (CINAHL).

#### Eligibility criteria

We used the Population-Concept-Context (PCC) framework for scoping reviews [29] to establish the eligibility criteria (see Table 1). The PCC framework is an adaptation for non-experimental research conceptually rooted in the PICO (population, intervention, comparison, outcome) [30] framework for identifying components of clinical evidence in systematic reviews.

**Table 1:** Eligibility criteria

Inclusion criteria	Exclusion criteria
**Population** Individuals, organizations, or community receiving TA services	• Articles published in languages other than English • Articles published before 2000 • Non-peer-reviewed articles • Peer-reviewed studies identified as a validation study, review, study protocol, trial registration, or any non-empirical study
**Concept** Peer-reviewed studies with a specific focus on the formative, process, or summative evaluation of TA that include both a description of the TA approach (activities or core elements) and TA output or outcomes data.	
**Context** The setting where TA is an intervention for capacity building or improvement.	

### Stage 3: Selecting studies

Our literature search strategy used the three-phase process outlined by the Joanna Briggs Institute [28]. First, we finalized the search strings and eligibility criteria. Then, we utilized Microsoft Excel to organize, deduplicate, and code articles. We employed a reference manager (EndNote X9) to extract and convert abstracts of relevant articles into a Microsoft Excel database. For study selection, research team members pilot screened 2% of article titles and abstracts from the five identified databases. During this process, two reviewers independently coded articles as “include,” exclude,” or “unsure, send to full-text review” using the eligibility criteria. The overall inter-rater reliability (IRR) was 0.90. Unresolved inter-rater discrepancies were presented to the research team for consensus coding. We used this initial pilot screening process to develop three screening questions:

- *Does the study objective indicate an evaluation of TA directly or of a program involving TA?*
- *Does the article include TA-specific outputs or outcomes?*
- *Does the article reflect the use of TA for systems-level capacity building/improvement?*

We then used the screening questions to identify the final list of articles.

### Stage 4: Charting the data

**Fig. 1:**
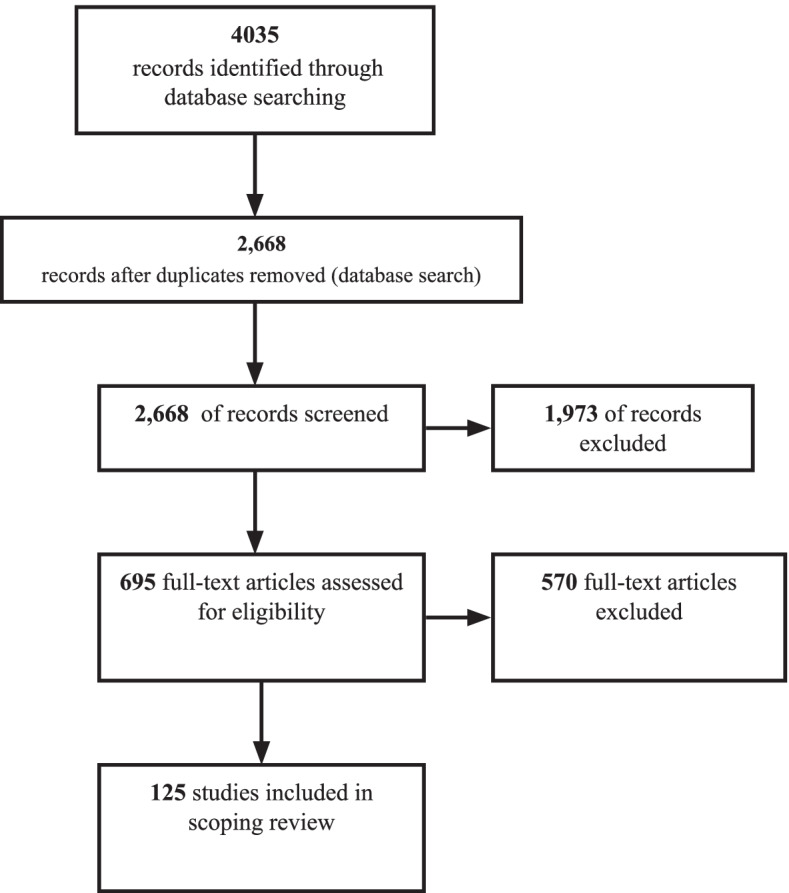
PRISMA flow diagram

We referred to the Preferred Reporting Items for Systematic and Meta-Analyses Extension for Scoping Reviews (PRISMA-ScRP): Checklist and Explanations guide [31] and the JBI qualitative data extraction instrument [28] to develop a standardized instrument for extracting information in accordance with the study research questions. Table 2 provides the categories that guided the coding of each article. We used an iterative process that involved piloting and refining the standardized form during the review of full-text articles. Four researchers (VS, ZJ, AM, and JK) reviewed, coded, and compared 10% of the articles to ensure coding consistency across dyads. Pairs of researchers (VS and ZJ; AM and JK) then independently reviewed and coded full-text articles using the eligibility criteria. Articles with discrepant dyad ratings were brought to the larger research team for a final decision. We used the PRISMA flow diagram model (Fig. 1) to report the final study inclusion and exclusion numbers.

**Table 2:** Data charting form: sample attributes

Study characteristics
First author’s last name Publication year Study location TA aim and activities Area of practice (e.g., child welfare, education)
TA evaluation attributes
Type of evaluation Measurement approach (e.g., survey, interview) Type of data (e.g., qualitative, quantitative) Data perspective (e.g., subjective, objective) TA outputs (e.g., dosage, reach) TA outcomes (e.g., individual, organization)

### Stage 5: Collating, summarizing, and reporting the results

In this stage, we prepared a quantitative and qualitative summary of data. The quantitative summary specifies the number of studies according to variables of interest (e.g., number or percentage of articles reporting TA outputs versus TA outcomes and number of articles utilizing each evaluation method identified). The qualitative summary is organized by the research questions. It includes an overview of concepts, describes the types of evidence available, and identifies themes and trends.

### Stage 6: Consulting with relevant stakeholders

The final stage of the scoping review involves consulting with relevant stakeholders to inform and validate the study findings. We utilized the consultative approach suggested by Peters and colleagues [28] to elicit feedback from experts and stakeholders throughout the study. Specifically, we discussed various topics throughout the scoping review, including the research questions, search terms, search criteria, target databases, data extraction variables, results, and study implications. Five subject matter experts, along with TA providers from the American Institute of Research (national TA center) and a Center of Excellence (Community Anti-Drugs Coalitions of America; CADCA) gave consultative feedback.

## Results

### Study characteristics

**Fig. 2:**
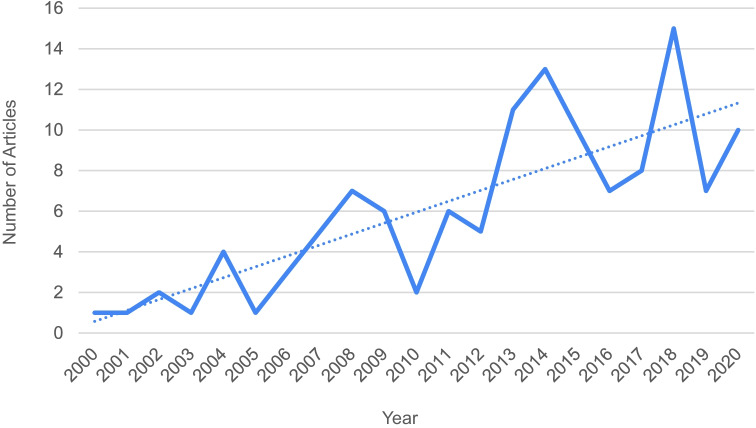
Trend line of TA articles published between January 2000 and June 2020

This scoping review includes 125 peer-reviewed articles published between January 2000 and June 2020 (see Fig. 2 and Additional file 1). The USA was the predominant study setting, representing 89% (*n*=112) of included articles. Study sample sizes ranged from 3 to 865,370, reflecting the number of participating individuals, programs, organizations, community coalitions, states, or countries. Approximately half of the studies (52%) used a descriptive research design. Other research designs included quasi-experimental (21%), experimental (13%), and correlational designs (13%). About 12% of studies explicitly defined technical assistance (see Table 3 for the definitions of TA provided).

**Table 3:** Definitions of technical assistance (TA) used in TA evaluation research studies

Article author(s) and year	Definition of technical assistance (TA)
Bonney et al. (2019) [32]	A multi-tiered approach to build the capacity of individuals or organizations to achieve substantial change (as cited in Fixsen, Blasé, Horner, and Sugai, 2009 [33]; Chilenski, Welsh, Olson, Hoffman, Perkins, and Feinberg, 2018 [34]).
Cerully et al. (2016) [35]	Support to help community-partner organizations execute their efforts (as cited in Mitchell, Florin, [36] and Stevenson, 2002 [37]).
Chiappone et al. (2018) [38]	TA is defined as targeted or tailored support given to an individual or organization to help assist with successful development, implementation, and evaluation of a program, policy, intervention, or service through shared knowledge, resources, and expertise (as cited in National Association for the Education of Young Children and National Association of Child Care Resource and Referral Agencies, 2011) [39].
Chilenski et al. (2018) [34]	TA involves external expertise and guidance designed to support the effective translation of EBIs into real-world settings (as cited in Forman, Olin, Hoagwood, Crowe & Saka, 2009 [12]; Wolff, 2001) [40].
Chilenski et al. (2016) [22]	TA, or the support and assistance that a prevention effort receives from someone or some organization that is not a part of a community team, has been theorized as very important in supporting high quality implementation of prevention programs specifically, and prevention systems more generally (as cited in Chinman et al., 2005 [14]; Forman, Olin, Hoagwood, Crowe, & Saka, 2009 [12]; Mitchell, Florin, & Stevenson, 2002 [36]; Wandersman & Florin, 2003 [41]; Wolff, 2001) [40].
Duffy et al. (2012) [42]	Individualized and hands-on intervention intended to address specific barriers in the context of a single individual or organization (as cited in Wandersman, Chien, & Katz, 2012 [10]).
Hunter et al. (2009) [43]	TA has been used to describe different types of activities, including community-friendly manuals, on-site consultation, regional workshops, train-the-trainers models, and interactive Web-based systems (as cited in Stevenson, Florin, Mills & Andrade, 2002).
Livet et al. (2018) [44]	Planned instructional activity to facilitate knowledge and skill acquisition (as cited in Leeman et al. 2015 [45]).
Moreland-Russell et al. (2018) [46]	For the purposes of this work, we considered “TA” for HPV and CRC as a multicomponent strategy consisting of in-person sessions supported by subject matter experts, facilitated development of action plans by state team members, and follow-up support calls which included webinars with team members and partners that were involved in the implementation of the specific activities in their respective action plans.
Olson, et al. (2020) [18]	TA has been defined as an individualized approach that provides implementation support to, and increasing capacity for, continuous quality improvement (CQI) among comparative effectiveness research. (as cited in Wandersman, Chien, and Katz, 2012 [10]; Chinman, Hunter, Ebener, et al., 2008 [47]).
Segre, O'Hara, Fisher (2013) [48]	TA consultations are sessions in which practitioners and host organizations gain the information, tools and support to implement new practices (as cited in Sullivan 1991 [49]).
Spadaro et al. (2011) [50]	Providing guidance, support, and expertise (as cited in Anderson, Bruner, & Satterfield, 1995) [51].
Rushovich et al. (2015) [52]	TA is a broad term that has been used to describe services that an outside entity provides to an agency or organization to help build its capacity to implement an innovation or improvement to their current operations (as cited in Sokol & Stiegert, 2010) [53].
Yazejian, Iruka (2015) [54]	On-site TA refers to any individualized professional development strategy that supports the application of skills to practice, such as coaching or professional development advising.
Young et al. (2020) [55]	The formal or informal engagement of an entity to one or more additional entities for the purpose of improving their capacity to accomplish their public health objectives (e.g., training, resources)

#### Applications of TA

Overall, the reasons for implementing TA were diverse (see Table 4). The most common reason for TA was to support the implementation of evidence-based practice or initiatives (41%). One-fifth (20%) of the articles indicated a combination of reasons. Evaluation capacity building (7%), coalition building (4%), improvement (4%), and workforce development (3%) were the next most cited reasons for TA. We aggregated less commonly noted reasons into a category entitled  “other” (21%), which included objectives such as needs assessment, knowledge sharing/dissemination, and tool development. TA was used in multiple areas of practice, with substance use, mental health, child welfare and youth development, public education, HIV prevention, and healthcare improvement most frequently noted.

**Table 4:** Reasons for and Frequency of Applications of TA

Characteristic	Frequency (percent)
**Reasons for implementing TA**	
Implement EBI	51 (41%)
Other (e.g., program development)	26 (21%)
Combination	25 (20%)
Evaluation capacity building	9 (7%)
Improvement	5 (4%)
Coalition building	5 (4%)
Workforce development	4 (3%)
**Area of practice**	
Other	46 (37%)
Substance use	18 (14%)
Mental health	15 (12%)
Public education	13 (10%)
Child welfare and youth development	13 (10%)
HIV prevention	11 (9%)
Healthcare improvement	7 (6%)
Housing	2 (2%)
**Type of TA**	
Combination	62 (49%)
Coaching	26 (21%)
Not specified	23 (18%)
Other	10 (8%)
Training	4 (3%)

Concerning the type of TA provided, nearly half of the studies (49%) involved a combination of TA activities (e.g., individual coaching, training, webinars, communities of practice). One-third of studies (32%) involved a singular TA activity (e.g., coaching, training, or other), and 18% of studies did not specify the type of TA provided.

### Research question 1: How has TA been evaluated in the scientific literature?

We examined Research question 1 through two questions, one regarding the methods used to measure TA and the other regarding the nature of TA outputs and outcomes. In the following section, we summarize the results from our scoping review.

#### RQ1a: What measurement approaches have been used to assess TA?

The majority of evaluation research studies were summative evaluations (72%). Process and formative evaluations were less common, comprising 15% of the studies jointly. Slightly over a tenth (13%) of studies employed a combination of the three types of evaluation.

A range of data collection methods was reported for measuring TA, including survey (26%), document review (16%), interview (15%), and observation (2%). The most common approach involved a combination of measurement methods (e.g., survey and document review) (38%).

Quantitative data were reported more frequently than qualitative data, 51% and 22%, respectively. A quarter of studies (26%) reported using both quantitative and qualitative TA data. Concerning data perspective, subjective data—such as respondents rating TA outcomes—were reported more frequently (42%) than objective data (21%, e.g., number of TA visits, availability of a comprehensive plan to address a need). Approximately two-fifths (37%) of studies reported both data perspectives. See Table 5 for a detailed summary of TA measurement approaches.

**Table 5:** Frequency of measurement approaches for assessing technical assistance

Characteristic	Frequency (percent)
**Type of evaluation**	
Summative	90 (72%)
Process	17 (14%)
Combination	16 (13%)
Formative	2 (1%)
**Method of measurement**	
Combination	47 (38%)
Survey	33 (26%)
Documentation review	20 (16%)
Interview	19 (15%)
Not reported	4 (3%)
Natural observation	2 (2%)
**Type of data**	
Quantitative	64 (51%)
Mixed methods	32 (26%)
Qualitative	28 (22%)
Not reported	1 (1%)
**Data perspective**	
Subjective	52 (42%)
Both	48 (37%)
Objective	26 (21%)

#### RQ1b: How have TA outputs and outcomes been conceptualized, and what are associated trends?

Outputs reflect the implementation of program activities that are directly salient to process and formative evaluation. In our scoping review, TA outputs were the activities or *mechanics of TA* delivery. The most frequently reported TA outputs were reach and modality. Available in 78% of studies, *reach* measures the number of units (e.g., individuals, organizations, etc) receiving TA.

*Modality* is the medium for TA delivery. Slightly over half of studies (54%) provided TA using a combination of mediums (e.g., in-person, phone, and virtual). In-person-only mediums (17%) were more common than phone/virtual exclusive modalities (6%).

*Cadence of contact* refers to the schedule of TA services (e.g., routine, and as-needed, fixed number) and was reported in 73% of the studies. A quarter of TA services were provided through a blended schedule involving routine and as-needed support. Aside from the blended schedule, as-needed (22%) service provision was more common than a routine (8%) or fixed-number service schedule (17%).

*Duration of engagement* reflects the total period of TA services, which is a broad indicator of dosage. As reported in 66% of studies, the duration of engagement ranged widely—from 2 days to 6 years. *Directionality* describes the source initiating TA contact (i.e., provider, recipient, or bi-directional). TA services were largely provider-initiated (21% proactive TA) or bi-directional (20%), and only 9% were recipient-initiated (reactive TA). Notably, half of the studies (50%) did not report directionality.

Lastly, *satisfaction* refers to feelings of fulfillment with TA and was reported in 18% of studies. Overall, respondents reported moderately high to high satisfaction with TA. In a small handful of studies where satisfaction was lower, recipients noted inadequate provider subject matter expertise, insufficient knowledge about the target setting, or inappropriate length of TA services (e.g., sessions too long or short). See Table 6 for a detailed summary of the TA outputs.

**Table 6:** Frequency of technical assistance output variables

Characteristic	Frequency (percent)
**Reach**	
Reported	98 (78%)
Not reported	27 (22%)
**Modality**	
Combination	68 (54%)
Not reported	29 (23%)
In-person	21 (17%)
Virtual/phone	7 (6%)
**Cadence**	
Not reported	34 (27%)
Combination	32 (26%)
As needed	28 (22%)
Fixed number	21 (17%)
Routinely	10 (8%)
**Duration of engagement**	
Reported	82 (66%)
Not reported	43 (34%)
**Directionality**	
Not reported	62 (50%)
Provider-initiated	27 (21%)
Bi-directional	25 (20%)
Recipient-initiated	11 (9%)
**Satisfaction with TA**	
Not reported	103 (82%)
Reported	22 (18%)

TA outcomes refer to the effect(s) or result(s) of TA services. TA outcomes were reported at the individual, programmatic/organizational, and community levels, and they included the use of both qualitative and quantitative data. Individual-level outcomes primarily related to behavioral change (19%), impact on knowledge (11%), and impact on skills (7%). All of the studies examining impact on knowledge reported that TA increased or improved recipient knowledge (e.g., [56–58]). Eighty-nine percent of studies examining impact on skills reported increased recipient skills associated with TA (e.g., [46, 59, 60]). Sixty-three percent of the studies examining behavior change (15 of 24 articles) reported a positive impact of TA (e.g., [61–63]). Other less frequently noted individual-level outcomes pertained to change in self-efficacy (4%), attitudes (2%), and motivation (2%).

Organizational-level outcomes were represented in 54% of studies, with 17% of these focused on particular programs within the organization. Overall, studies indicate a positive association between the use of TA and organizational-level outcomes, particularly concerning performance or service delivery quality (e.g., [64–66]), program/EBP implementation (e.g., [18, 56, 57, 62, 67–69]), evaluation capacity [70–74], and collaboration among stakeholders [46, 57, 75].

Studies reporting on the differential impact of TA attributed variations to organizational size, age, staff experience, staff buy-in, and availability of financial incentives for participation. For example, one study indicated that larger firms are more likely to report increased market share, sales, and profits due to TA compared to smaller firms [76]. Another study reported that better healthcare quality was associated with healthcare providers who did not receive financial incentives and TA compared to an incentivized group [77], raising questions about the value of supplementing TA with extrinsic rewards.

Several studies examined the relationship between TA dosage (number of TA hours or calls) and organizational-level outcomes. Most of these studies reported positive findings (e.g., [66, 78, 79]). However, two studies reported no association [42, 80]. One study [81] reported both significant and nonsignificant associations, which varied by capacity areas examined (e.g., evaluation, sustainability).

Community-level interventions pertain to capacity building efforts in a geographically defined area(s) such as a city, county, region, state, providence, or country. Community-level outcomes were reported in 16% of the studies, most relating to child welfare and HIV prevention. Sample outcomes included associations between TA dose and pandemic preparedness [82], community readiness and levels of collaboration [34], TA and collaboration level or team functioning [22, 83, 84], and TA and service or program quality [64, 85]. Results largely reflected partial gains in community capacity (e.g., public health preparedness, development of a plan of collaborative agreement, access to resources, and partnerships). Commonly cited limitations within community-level studies were small sample size, limited generalizability, and lack of a control group. These articles also tended to be scarce in descriptions about TA (e.g., activities and reach). See Table 7 for a detailed summary of TA outcomes.

**Table 7:** Frequency of individual, organizational, and community level outcomes

Characteristic	Frequency (percent)
**Individual level outcomes**	
**Behavior**	
Increased	15 (12%)
Decreased	7 (6%)
No change	2 (1%)
Not reported	101 (81%)
**Knowledge**	
Increased	14 (11%)
Not reported	111 (89%)
**Skills**	
Increased	8 (6%)
No change	1 (1%)
Not reported	116 (93%)
**Self-efficacy**	
Increased	5 (4%)
Decreased	1 (1%)
Not reported	119 (95%)
**Motivation**	
Increased	3 (2%)
Not reported	122 (98%)
**Awareness**	
Increased	2 (2%)
Not reported	123 (98%)
**Attitudes**	
Increased	2 (2%)
Not reported	123 (98%)
**Organizational level outcomes**	
Organizational program	21 (17%)
Combination of outcomes	15 (12%)
Staff capacities	5 (4%)
Organizational structure	3 (2%)
Resource utilization	3 (2%)
Other	20 (16%)
Not reported	58 (46%)
**Community level outcomes**	
Reported	20 (16%)
Not reported	105 (84%)

### Research question 2: To what extent has TA provision resulted in sustainable improvements in organizations and communities?

We defined “sustainable improvements” as positive changes resulting from TA that were maintained beyond the period of TA services. The degree to which gains associated with TA are sustained over time was reported in 5% of studies. In these cases, improvements associated with TA were largely not sustained, with the effects of TA disappearing after a period of time (e.g., 1 year). One experimental study [86] found that gains associated with TA did not sustain except for the group that received the greatest dose of implementation support (i.e., general training and TA). Leadership engagement and staff commitment were identified as critical to sustaining gains associated with TA [87]. Additionally, recipients noted the importance of ongoing TA for sustaining improvements.

## Discussion

This scoping review draws on two decades of peer-reviewed publications to summarize the evidence on the evaluation and effectiveness of TA. Findings suggest that TA can effectively build system capacity across diverse settings to enhance implementation. As a capacity building strategy, TA is often delivered to organizations serving socially vulnerable (e.g., persons with serious mental illness, addiction, and HIV) and under-resourced populations. TA delivery to programs supporting vulnerable populations holds promise for advancing health equity and social justice. The increasing number of published articles per year over the two decades signals a growing recognition, application, and reporting of TA.

Knowing how well TA is implemented, which features of TA are most successful for capacity building, and the overall effectiveness of TA relies on quality evaluation research. Although a critical appraisal of the quality of evaluative research on TA was not a focus of this review, we would be remiss if we did not acknowledge overarching methodological gaps that limit our ability to draw meaningful insights across TA literature. Findings from our scoping review support assertions that TA delivery rarely involves systematic planning, implementation, and evaluation methods [13, 23]. Further, we encountered a general lack of definitional clarity, rigorous evaluation research designs, and effective reporting standards in the literature. Increasing transparency and reporting quality of TA research is essential for maximizing impact. In the following sections, we reflect on four aspects core to enhancing the evaluation of TA: defining, designing, measuring, and reporting TA. See Table 8 for a summary of recommendations for enhancing each of these four areas.

**Table 8:** Summary of recommendations to advance the evaluation and effectiveness of TA

Main Insight	Recommendation
1. **Need for a Standard Definition of TA**	Use a consensus method (e.g., Delphi Technique) which includes a panel of expert TA practitioners, researchers, and recipients to develop a standard definition of TA. Consider the following defining features of TA when establishing a standard definition: ○ Aim is to increase capacity ○ Services target the systems-level (organization, community) ○ Supports are targeted and tailored ○ Supports are provided by a subject matter expert or specialist
2. **Need for More Robust and Rigorous Evaluation Research Designs**	• Use more robust evaluation research designs (e.g., experimental designs) to identify causal links between TA implementation and outcomes. • Increase use of longitudinal study designs to understand the sustainability of TA. Include control and matching techniques to compare outcomes over time. • Consider approaches rooted in design research (formative experiments occurring in real-world settings) to examine downstream effects of TA.
3. **Need for More Reliable Measures and Objective Measures of TA Processes and Outcomes**	• Use self-report measures to assess TA recipient attitudes and beliefs, particularly regarding TA satisfaction, self-efficacy, and commitment to change. • Prioritize the use of objective data to measure outcomes about knowledge, skills, behavior change, and system-level changes. • When feasible, use a mixed-methods approach to capture subjective and objective data to enable data triangulation. • Develop and use psychometrically sound instruments to assess TA.
4. **Need for Reporting Standards**	• Use a TA logic model to guide the systematic documentation of TA inputs, processes, outputs, and outcomes. • Develop reporting standards for TA evaluation research studies. Consider the following items for a reporting checklist: ○ Provide an explicit conceptual and operational definition for TA. Upon availability, utilize a standard TA definition. ○ State the specific aim(s) and targeted direct and indirect outcomes for utilizing TA (e.g., to implement an evidence-based practice/intervention, coalition building, workforce development). ○ Provide detailed descriptions of TA activities (e.g., coaching, training, tools, combination), including data relating to core mechanics of TA (e.g., modality, reach, duration of engagement, directionality, frequency of contact). Additionally, describe the methods of measuring TA activities (e.g., measurement tools, procedures). ○ Where possible, report: i) the effect of specific TA activities to disaggregate attributions, in addition to the total effect, ii) both direct and indirect outcomes of TA and iii) longitudinal outcomes.

### Main insight 1: A need for a standard definition of TA

Our synthesis indicates two significant definitional limitations in evaluation studies of TA. First, studies rarely include an explicit TA definition (only 12% of examined studies). Second, among studies that do include TA definitions, definitions are highly variable, reflecting different understandings of TA’s purpose, process, and provision. For example, some definitions reflect a general aim for TA (e.g., “to build the capacity of individuals or organizations” [32]), while other definitions offer a more specific aim (e.g., “to facilitate knowledge and skill acquisition” [44]). In terms of implementation, some TA definitions encompass a variety of processes or activities (e.g., a “multi-tiered approach” [32]; “different types of activities including community-friendly manuals, on-site consultation, regional workshops, train-the-trainers models, and interactive Web-based systems” [43]). Others use less specific language (e.g., “support to help…” [36]; “tailored or targeted support to…” [38]). Relatively few definitions reference who is providing TA (e.g., “external expertise” [12]; “an outside entity” [52]). While these differences may appear semantic or inconsequential, lacking a consistent definition of TA creates challenges for identifying relevant research and best-practices, and it reduces comparability across studies.

Perhaps the most significant challenge is simply identifying reliable standards of what is and is not TA. Specifically, *what are the necessary and sufficient conditions (purposes, activities, and processes) that allow researchers or practitioners to claim TA practice?* For example, *is TA practice inclusive of training? If so, in what instances and why?* Relatedly, *what do we mean by “tailored” and “targeted” services? And when non-tailored resources (e.g., informational websites and, guides) are provided to all recipients - sometimes referred to as “universal” services - is it appropriate to conceptualize those activities as part of TA?* Additionally, *when is it appropriate to evoke TA terminology over alternative terminologies such as coaching, consulting, or counseling which also address capacity building? Are members internal to an organization who provide capacity building supports considered TA providers, or are TA providers inherently external to an organization?* These questions are foundational to reliable and valid TA measurement.

To disentangle these complex questions and establish a standard definition for TA, we suggest a consensus method, such as the Delphi technique [88], with a panel of expert TA practitioners, researchers, and TA recipients. It may be useful to identify overlapping features across existing definitions that can serve as a foundation for future consensus. Drawing from the 15 definitions offered in our synthesis, we observed the following defining features of TA:

- Aim is to increase capacity
- Services target the systems-level (e.g., organization, community)
- Supports are individualized (i.e., targeted/tailored)
- Supports are provided by a subject matter expert or specialist

These characteristics can serve as a starting place for developing a reliable, standard definition for TA.

### Main insight 2: A need for more robust and rigorous evaluation research designs

More robust evaluation research designs are needed to (i) establish causal relationships between TA implementation and outcomes; (ii) understand the sustainability of TA outcomes, including what contributes to sustained outcomes; and (iii) elucidate the direct and indirect impact of TA. In our scoping review, we observed a reliance on descriptive methodologies (52%) and modest use of experimental designs (13%). While descriptive studies have merit, particularly in explaining the process of TA delivery, experimental designs can identify causal links between TA implementation and outcomes. For example, one relationship that has been examined but remains inconclusive is between TA intensity (i.e., dose and degree of tailoring) and gains in capacity. Some studies have reported a positive relationship (e.g., [66, 78, 79]), while other studies indicate no significant relationship (e.g., [42, 80]). The relationship between TA intensity and outcomes warrants further research and is best examined using experimental approaches to establish causality.

We found that only 5% of the scoping review studies examined the sustainability of TA outcomes. Longitudinal study designs, including baseline measures of dependent variables, are essential to understanding which TA outcomes are sustainable over time and for how long. This understanding is essential for funders, researchers, and practitioners evaluating expected returns on current or future TA investments. However, several threats to validity warrant particular attention when measuring impact over time, including maturation, effects of history, instrumentation, selection, attrition, and regression to the mean. We encourage future research to pursue longitudinal studies, including control groups or matching techniques, to compare TA outcomes over time.

Lastly, the majority of evaluation studies have been designed to examine the direct impact of TA on recipient systems, such as changes in organizational capacity for implementing an evidence-based practice or staff capacity. The downstream impact of TA has received less attention, perhaps due to inherent measurement challenges associated with conducting evaluation research in complex settings. According to the *Interactive Systems Framework for Dissemination and Implementation* (ISF), TA is a support system element designed to build delivery system capacity, which, in turn, enhances implementation toward a set of desired outcomes [24, 89]. As such, intervention (e.g., programmatic) outcomes are most appropriately monitored and measured in relation to the delivery system—that is, the setting/system receiving direct TA services. However, evaluation research designs that examine both direct and indirect outcomes of TA are needed to better understand both immediate benefits of TA to the capacity of the delivery system and downstream benefits of TA, including what intervention outcomes can be appropriately attributed to TA. Design research may be a useful approach for systematically examining downstream effects of TA when TA involves multiple activities (e.g., coaching, training, and communities of practice). Originating in the field of education, this approach involves developing formative experiments to test and refine interventions occurring in real-world rather than controlled settings [90–92]. Unlike hypothesis testing, which targets a limited number of variables, design research examines all aspects of an intervention to develop a profile of the intervention in practice. In this way, the most effective components or characteristics of TA for a particular setting and target population can be determined.

### Main insight 3:  A need for more reliable and objective measures of TA processes and outcomes

The scoping review revealed that subjective data (e.g., self-ratings of change in knowledge resulting from TA) was reported roughly twice as often as objective data (e.g., knowledge-based assessment). Further, less than half of the studies included subjective and objective TA data. Subjective data are valuable for understanding recipient engagement, which can serve as one indicator of increased capacity [70]. Self-report data can also offer ease and efficiency for assessing TA outcomes [76]. However, self-report data are subject to social desirability and reference bias and may not reflect an actual change in knowledge or skills.

We recommend that researchers utilize self-report measures to assess recipient attitudes and beliefs, particularly regarding TA satisfaction, self-efficacy, and commitment to change. For outcomes about knowledge, skills, behavior change, and change in system-level policies and practices, we suggest prioritizing objective data, such as a knowledge assessment, demonstration of skill-based competencies, or tangible observations of practice change. When feasible, a mixed-methods approach that captures subjective and objective data is optimal, allowing for data triangulation. For instance, Clark et al. [67] utilized a mixed-methods approach to measuring TA outcomes by employing observational assessments of TA recipients’ teaching skills and using structured interviews. Similarly, Chinman et al. [80] measured the adherence, quality, and dosage of TA and used a program performance interview.

Specifically, in relation to surveys, we observed the absence of a widely used, psychometrically validated, and reliable instrument for assessing TA implementation and effectiveness. These measures exist for related capacity building strategies (e.g., training and communities of practice). Developing psychometrically sound instruments for assessing TA is a critical step toward enhancing measurement validity and reliability. An instrument to assess TA effectiveness might reflect two broad process constructs: TA techniques (e.g., responsive, client-centric, and proactive) and the TA relationship (e.g., trust, collaboration, communication [13]), and also include items assessing TA outputs and outcomes.

### Main insight 4: A need for reporting standards

Widespread variation in the reporting of TA implementation, measurement, and outcomes constrained our ability to draw insights across studies. Our findings suggest that a majority of reported TA outcomes cannot be directly attributed to a specific TA activity or hierarchy of activities. Nearly half of the studies we examined included two or more TA activities (e.g., individualized coaching and training; process frameworks such as Getting To Outcomes®), often in a single measure of TA. Another 18% did not describe the activities that constituted TA. This failure to consistently describe and isolate TA activities makes it difficult to determine how particular TA practices produce positive outcomes. Undoubtedly, the heterogeneity of the reporting of TA is a byproduct of the diverse definitions for TA—linking back to Main insight 1. As a result, practitioners may be overinvesting in ineffective activities or underinvesting in effective activities (e.g., providing individual coaching when expert training is sufficient to produce outcomes). Moreover, a lack of reporting clarity severely limits practitioners’ ability to replicate positive findings. Studies that fail to meaningfully describe a TA intervention (e.g., modality, dosage, cadence, duration, and reach) may prohibit the scaling of effective interventions. For the TA research literature to meaningfully contribute to effective TA practice, it must articulate a clear explanation of which TA activities make an impact, how and when this happens, and for how long the impact occurs. We have developed a *Logic Model for TA Effectiveness* that we use in our practice as a skeletal frame to guide TA planning, implementation, and evaluation (available via the corresponding author). This logic model specifies the theory of change for a set of TA activities using the domains of inputs, processes, outputs, and outcomes. The *Logic Model for TA Effectiveness* may be a valuable tool for developing reporting standards.

Lastly, we recommend collective investment from funders, authors, reviewers, and editors in developing minimum reporting standards for TA evaluation research studies and that TA recipients and providers participate in the process. We offer the following reporting checklist as a starting point:

- Provide an explicit conceptual and operational definition for TA, and upon availability, utilize a standard definition.
- State the specific aim(s) and targeted direct and indirect outcomes for utilizing TA (e.g., to implement an evidence-based practice/intervention, coalition building, and workforce development).
- Provide detailed descriptions of TA activities (e.g., coaching, training, and tools, or a combination of these), including data relating to core mechanics of TA (e.g., modality, reach, duration of engagement, directionality, and frequency of contact). Additionally, describe the methods of measuring TA activities (e.g., measurement tools and procedures).
- Where possible, report (i) the effect of specific TA activities to disaggregate attributions, in addition to the total effect; (ii) both direct and indirect outcomes of TA; and (iii) longitudinal outcomes.

Consistent reporting of TA interventions and outcomes will help build the theory of change for TA.

### Study limitations

While we sought to be comprehensive with this review, our search parameters may have missed evaluation research articles. Our search strategy included five interdisciplinary databases where TA literature is commonly published. A search of other bibliographic databases may yield other relevant studies. Further, we limited searches within each database to peer-reviewed articles, potentially skewing data toward academic research and away from practice. We conducted a pilot search to establish 12 search terms to identify relevant studies. Although we used an iterative process to determine the final set of search terms, other key terms may exist that are linked to articles not identified in our search. Additionally, we reported that most evaluation research studies involved TA delivered in the USA. This trend could be a byproduct of limiting articles to English (the language of the research team). Including articles published in other languages would plausibly reveal a broader set of studies. Lastly, we coded only outcomes that were explicitly associated with TA. Articles that bundled TA outcomes with other capacity building outcomes were excluded when clear attributions to TA outcomes could not be delineated. As such, this scoping review is a conservative representation of the number of evaluation studies involving TA.

This study aimed to describe the TA evaluation literature rather than formally assess the quality of TA evaluation. Like research across any topic, the breadth and depth of methodological descriptions were highly variable across studies. We summarize TA methods and findings as they were reported in each article, regardless of reporting quality. We did not seek out new information or clarification from the authors. As such, study methods may be more robust in practice than they appear in our results. We encourage authors to adhere to reporting standards that will advance the study, practice, and theory of TA.

Although this scoping review includes two decades of evaluation research, it primarily reflects findings from studies published prior to the COVID-19 pandemic. Pandemic responses may have a lasting impact on TA provision. In fact, telework is forecasted to remain a sustained fixture across industries [93, 94]. As such, we anticipate that exclusively virtual TA will play a more prominent role in the immediate future of TA than findings from this review may suggest.

## Conclusion

TA is a time and resource-intensive approach to organizational and community capacity building that has grown in use across diverse settings over the past two decades. Advances in the science and practice of TA hinge on understanding which aspects of TA are effective, and when, how, and for whom these aspects of TA are effective. Addressing these core questions requires (i) a widely adopted standard definition for TA; (ii) more robust and rigorous evaluation research designs that involve comparison groups and assessment of direct, indirect, and longitudinal outcomes; (iii) increased use of reliable and objective measures of TA outcomes; and (iv) the development of reporting standards. We view this scoping review as a foundation for improving the state of the science and practice of evaluating TA.

## Supplementary Information

**Additional file 1.** Summary of articles included in scoping review.

## Data Availability

The datasets used during the current study are available from the corresponding author on request.

## Abbreviations

TA: Technical assistance
EBP: Evidence-based practice
EBSIS: Evidence-based System for Innovation Support
RQ: Research question
PCC: Population-Concept-Context
PICO: Population, Intervention, Comparison, Outcome
IRR: Inter-rater reliability
PRISMA-ScRP: Preferred Reporting Items for Systematic and Meta-Analyses Extension for Scoping Reviews
CQI: Continuous Quality Improvement
ISF: Interactive Systems Framework for Dissemination and Implementation
