# Supplementary material for: A scoping review of the evaluation and effectiveness of technical assistance
Source: Implement Sci Commun. 2022 Jun 28;3:70. doi: 10.1186/s43058-022-00314-1 (PMC9238031; doi:10.1186/s43058-022-00314-1)
Supplement: Supplementary file 1 — Additional file 1. Summary of articles included in scoping review. [file 43058_2022_314_MOESM1_ESM.docx]

Appendix. Summary of articles included in scoping review

| Reference | Location | Sample | Area of Practice | Type of Evaluation | Method of Measurement |
| --- | --- | --- | --- | --- | --- |
| Abraham, A. J., Andrews, C. M., Grogan, C. M., Pollack, H. A., D’Aunno, T., Humphreys, K., & Friedmann, P. D. (2018). State-targeted funding and technical assistance to increase access to medication treatment for opioid use disorder. *Psychiatric services*, *69*(4), 448-455. | US-General | 456 non-OTP programs | Substance Use | Summative | Survey |
| Abraham, A. J., Smith, B. T., Andrews, C. M., Bersamira, C. S., Grogan, C. M., Pollack, H. A., & Friedmann, P. D. (2019). Changes in state technical assistance priorities and block grant funds for addiction after ACA implementation. *American journal of public health*, *109*(6), 885-891. | US-General | 2014 n = 50 individuals and 2017 n = 49 individuals. | Substance Use | Summative | Survey |
| Arevian, A. C., Jones, F., Tang, L., Sherbourne, C. D., Jones, L., Miranda, J., & Community Partners in Care Writing Group. (2019). Depression remission from community coalitions versus individual program support for services: findings from Community Partners in Care, Los Angeles, California, 2010–2016. *American Journal of Public Health*, *109*(S3), S205-S213. | US- City | 283 individuals | Mental Health | Summative | Survey |
| Asada, Y., Gilmet, K., Welter, C., Massuda-Barnett, G., Kapadia, D. A., & Fagen, M. (2019). Applying theory of change to a structural change initiative: Evaluation of model communities in a diverse county. *Health Education & Behavior*, *46*(3), 377-387. | US-Single State | 6 model communities | Other | Summative | Interview |
| Baker, H. M., Ranney, L. M., & Goldstein, A. O. (2016). Pilot implementation of a wellness and tobacco cessation curriculum in north carolina group homes. *Community mental health journal*, *52*(4), 433-438. | US-Single State | 2 group home facilities | Substance Use | Combination | Interview |
| Baldwin, C. K., & Wilder, Q. (2014). Inside quality: Examination of quality improvement processes in afterschool youth programs. *Child & Youth Services*, *35*(2), 152-168. | US-General | 3 afterschool programs | School | Summative | Combination |
| Barwick, M. A., Urajnik, D. J., & Moore, J. E. (2014). Training and maintaining system-wide reliability in outcome management. *Journal of Child and Family Studies*, *23*(1), 85-94. | Non-US-Region | 315 external TA group; 140 internal TA group | Mental Health | Summative | Interview |
| Beach, L. B., Reidy, E., Marro, R., Johnson, A. K., Lindeman, P., Phillips, G., ... & Greene, G. J. (2020). Application of a Multisite Empowerment Evaluation Approach to Increase Evaluation Capacity Among HIV Services Providers: Results From Project Pride in Chicago. *AIDS Education and Prevention*, *32*(2), 137-S5. | US-Single State | 41 individuals | HIV | Summative | Survey |
| Bellei, C. (2013). Supporting instructional improvement in low-performing schools to increase students’ academic achievement. *The journal of educational research*, *106*(3), 235-248. | Non-US-City | 69 primary schools | School | Summative | Survey |
| Beuermann, D. W., & Amelina, M. (2018). Does participatory budgeting improve decentralized public service delivery? Experimental evidence from rural Russia. *Economics of Governance*, *19*(4), 339-379. | Non-US-Multi-Region | 109 settlements | Other | Summative | Combination |
| Boas, S. J., Bishop, T. F., Ryan, A. M., Shih, S. C., & Casalino, L. P. (2014, July). Electronic health records and technical assistance to improve quality of primary care: lessons for regional extension centers. In *Healthcare* (Vol. 2, No. 2, pp. 103-106). Elsevier. | US-Single State | 17 physicians | EHR | Process | Interview |
| Bonney, T., Welter, C., Jarpe-Ratner, E., & Conroy, L. M. (2019). Understanding the role of academic partners as technical assistance providers: results from an exploratory study to address precarious work. *International Journal of Environmental Research and Public Health*, *16*(20), 3903. | US-Single State | 22 individuals | Other | Combination | Combination |
| Braciszewski, J. M., Olson, B. D., Jason, L. A., & Ferrari, J. R. (2006). Chapter 5: The Influence of Policy on the Differential Expansion of Male and Female Self-Run Recovery Settings. *Journal of Prevention & Intervention in the Community*, *31*(1-2), 51-62. | US-  General | 568 individuals | Housing | Summative | Documentation Review |
| Bruder, M. B., Dunst, C. J., Wilson, C., & Stayton, V. (2013). Predictors of confidence and competence among early childhood interventionists. *Journal of Early Childhood Teacher Education*, *34*(3), 249-267. | US- Multi State | 1668 individuals | Other | Summative | Survey |
| Buller, D. B., Bettinghaus, E. P., Helme, D., Young, W. F., Borland, R., Maloy, J. A., ... & Walther, J. B. (2011). Supporting tobacco control: Stimulating local newspaper coverage with a technical assistance website for local coalitions. *Health Promotion*  *Practice*, *12*(6_suppl_2), 186S-194S. | US-Single State | 24 coalitions; 73 newspapers | Substance Use | Summative | Documentation Review |
| Campbell, R., Townsend, S. M., Shaw, J., Karim, N., & Markowitz, J. (2015). Can a workbook work? Examining whether a practitioner evaluation toolkit can promote instrumental use. *Evaluation and Program Planning*, *52*, 107-117. | US-General | 6 programs | Other | Combination | Combination |
| Camponeschi, J., Vogt, C. M., Creswell, P. D., Mueller, M., Christenson, M., & Werner, M. A. (2017). Taking action with data: improving environmental public health at the community level. *Journal of Public Health Management and Practice*, *23*, S72-S78. | US-Single State | 8 local health departments | Other | Combination | Combination |
| Castillo, E. G., Shaner, R., Tang, L., Chung, B., Jones, F., Whittington, Y., ... & Wells, K. B. (2018). Improving depression care for adults with serious mental illness in underresourced areas: community coalitions versus technical support. *Psychiatric services*, *69*(2), 195-203. | US- City | 504 individuals | Mental Health | Summative | Survey |
| Cerully, J. L., Collins, R. L., Wong, E. C., & Yu, J. (2016). The Mental Health Association of San Francisco Partner Organizations Meet Their Goals in Stigma Reduction Efforts: Results of a Qualitative Evaluation of the Technical Assistance Process. *Rand Health Quarterly*, *5*(3). | US-Single State | 15 community partners | Mental Health | Process | Interview |
| Chaple, M., & Sacks, S. (2016). The impact of technical assistance and implementation support on program capacity to deliver integrated services. *The Journal of Behavioral Health Services & Research*, *43*(1), 3-17. | US-Single State | 120 outpatient programs | Mental Health | Summative | Combination |
| Chiappone, A., Smith, T. M., Estabrooks, P. A., Rasmussen, C. G., Blaser, C., & Yaroch, A. L. (2018). Technical assistance and changes in nutrition and physical activity practices in the National Early Care and Education Learning Collaboratives Project, 2015–2016. *Preventing chronic disease*, *15*, E47. | US- Multi State | 84 programs | Child Welfare | Summative | Combination |
| Chilenski, S. M., Welsh, J., Olson, J., Hoffman, L., Perkins, D. F., & Feinberg, M. E. (2018). Examining the highs and lows of the collaborative relationship between technical assistance providers and prevention implementers. *Prevention Science*, *19*(2), 250-259. | US- Multi State | 14 communities | Child Welfare | Summative | Combination |
| Chilenski, S. M., Perkins, D. F., Olson, J., Hoffman, L., Feinberg, M. E., Greenberg, M., ... & Spoth, R. (2016). The power of a collaborative relationship between technical assistance providers and community prevention teams: A correlational and longitudinal study. *Evaluation and Program Planning*, *54*, 19-29. | US- Multi State | 14 communities | Other | Summative | Combination |
| Chinman, M., Hunter, S. B., Ebener, P., Paddock, S. M., Stillman, L., Imm, P., & Wandersman, A. (2008). The getting to outcomes demonstration and evaluation: an illustration of the prevention support system. *American journal of community psychology*, *41*(3), 206-224. | US-General | 2 community-based prevention coalitions | Substance Use | Combination | Documentation Review |
| Chinman, M., Hannah, G., & McCarthy, S. (2012). Lessons learned from a quality improvement intervention with homeless veteran services. *Journal of Health Care for the Poor and Underserved*, *23*(3), 210-224. | US-Region | 54 staff members participated in study, 32 staff members received TA | Other | Summative | Combination |
| Chinman, M., Acosta, J., Ebener, P., Burkhart, Q., Malone, P. S., Paddock, S. M., ... & Tellett-Royce, N. (2013). Intervening with practitioners to improve the quality of prevention: one-year findings from a randomized trial of assets-getting to outcomes. *The Journal of Primary Prevention*, *34*(3), 173-191. | US-Single State | 12 prevention coalitions | Other | Summative | Documentation Review |
| Chinman, M., Acosta, J., Ebener, P., Malone, P. S., & Slaughter, M. E. (2015). Can implementation support help community-based settings better deliver evidence-based sexual health promotion programs? A randomized trial of Getting To Outcomes®. *Implementation Science*, *11*(1), 1-16. | US- Multi State | 32 Boys and Girls Clubs | Child Welfare | Summative | Combination |
| Choi, J. H., McCart, A. B., Hicks, T. A., & Sailor, W. (2019). An analysis of mediating effects of school leadership on MTSS implementation. *The Journal of Special Education*, *53*(1), 15-27. | US-General | 59 schools | School | Summative | Documentation Review |
| Clark, N. M., Cushing, L. S., & Kennedy, C. H. (2004). An intensive onsite technical assistance model to promote inclusive educational practices for students with disabilities in middle school and high school. *Research and Practice for Persons with Severe Disabilities*, *29*(4), 253-262. | US-General | 3 teachers | School | Summative | Combination |
| Coleman, K., Phillips, K. E., Van Borkulo, N., Daniel, D. M., Johnson, K. E., Wagner, E. H., & Sugarman, J. R. (2014). Unlocking the black box: supporting practices to become patient-centered medical homes. *Medical care*, S11-S17. | US-General | 65 safety net sites | Other | Combination | Survey |
| Compton, D. W., MacDonald, G., Baizerman, M., Schooley, M., & Zhang, L. (2008). Using evaluation capacity building (ECB) to interpret evaluation strategy and practice in the United States National Tobacco Control Program (NTCP): A preliminary study. *The Canadian Journal of Program Evaluation*, *23*(3), 199. | US- Multi State | 5 states | Substance Use | Summative | Combination |
| Dancy-Scott, N., Williams-Livingston, A., Plumer, A., Dutcher, G. A., & Siegel, E. R. (2016). Enhancing the capacity of community organizations to evaluate HIV/AIDS information outreach: A pilot experiment in expert consultation. *Information services & use*, *36*(3-4), 217-230. | US-General | 4 ACIOP awardees | HIV | Summative | Combination |
| Duffy, J. L., Prince, M. S., Johnson, E. E., Alton, F. L., Flynn, S., Faye, A. M., ... & Hinzey, A. L. (2012). Enhancing teen pregnancy prevention in local communities: Capacity building using the interactive systems framework. *American Journal of Community Psychology*, *50*(3), 370-385. | US-General | 14 partner organizations | Other | Summative | Combination |
| Elliott, D. S., & Mihalic, S. (2004). Issues in disseminating and replicating effective prevention programs. *Prevention Science*, *5*(1), 47-53. | US-General | 42 sites | Other | Summative | Not Reported |
| Emmons, K. M., Geller, A. C., Viswanath, V., Rutsch, L., Zwirn, J., Gorham, S., & Puleo, E. (2008). The SunWise Policy intervention for school-based sun protection: a pilot study. *The Journal of School Nursing*, *24*(4), 215-221. | US-Single State | 28 schools | School | Summative | Survey |
| Emshoff, J., Blakely, C., Gray, D., Jakes, S., Brounstein, P., Coulter, J., & Gardner, S. (2003). An ESID case study at the federal level. *American Journal of Community Psychology*, *32*(3-4), 345-357. | US-General | 16 sites | Substance Use | Summative | Combination |
| Farrell, A. F., Collier‐Meek, M. A., & Furman, M. J. (2019). Supporting out‐of‐school time staff in low resource communities: A professional development approach. *American journal of community psychology*, *63*(3-4), 378-390. | US-Region | 3 Out-of-School Time programs | School | Summative | Natural Observation |
| Feinberg, M. E., Ridenour, T. A., & Greenberg, M. T. (2008). The longitudinal effect of technical assistance dosage on the functioning of Communities That Care prevention boards in Pennsylvania. *The Journal of Primary Prevention*, *29*(2), 145-165. | US-Single State | 2003-2005: 570, 867, 819 individuals | Other | Summative | Survey |
| Furukawa, M. F., King, J., & Patel, V. (2015). Physician attitudes on ease of use of EHR functionalities related to meaningful use. *Am J Manag Care*, *21*(12), e684-e692. | US-General | 1793 individuals | EHR | Summative | Survey |
| Gibbs, D. A., Hawkins, S. R., Clinton-Sherrod, A. M., & Noonan, R. K. (2009). Empowering programs with evaluation technical assistance. *Health Promotion Practice*, *10*(1_suppl), 38S-44S. | US-General | 7 participants | Other | Summative | Interview |
| Gibbs, D., Napp, D., Jolly, D., Westover, B., & Uhl, G. (2002). Increasing evaluation capacity within community-based HIV prevention programs. *Evaluation and program Planning*, *25*(3), 261-269. | US-General | 61 CBOs, nine health departments, and 28 technical assistance providers | HIV | Summative | Interview |
| Gothro, A., Hanno, E. S., & Bradley, M. C. (2020). Challenges and solutions in evaluation technical assistance during design and early implementation. *Evaluation Review*, 0193841X20911527. | US-General | 18 communities (YARH) & 9 cohorts (P3) | Child Welfare | Summative | Not Reported |
| Grisham-Brown, J., Hallam, R. A., & Pretti-Frontczak, K. (2008). Preparing Head Start personnel to use a curriculum-based assessment: An innovative practice in the “age of accountability”. *Journal of Early Intervention*, *30*(4), 271-281. | US-Single State | 6 individuals; 14 individuals | School | Summative | Not Reported |
| Gross, J. M., McCarthy, C. F., Verani, A. R., Iliffe, J., Kelley, M. A., Hepburn, K. W., ... & Riley, P. L. (2018). Evaluation of the impact of the ARC program on national nursing and midwifery regulations, leadership, and organizational capacity in East, Central, and Southern Africa. *BMC Health Services Research*, *18*(1), 1-11. | Non-US-Multi-Region | 17 individuals | HIV | Summative | Documentation Review |
| Gutin, S. A., Amico, K. R., Hunguana, E., Munguambe, A. O., & Rose, C. D. (2017). The relationship of repeated technical assistance support visits to the delivery of positive health, dignity, and prevention (PHDP) messages by healthcare providers in Mozambique: A longitudinal multilevel analysis. *Journal of the International Association of Providers of AIDS Care (JIAPAC)*, *16*(5), 487-493. | Non-US-Country | 153 healthcare providers | HIV | Summative | Combination |
| Honeycutt, S., Carvalho, M., Glanz, K., Daniel, S. D., & Kegler, M. C. (2012). Research to reality: a process evaluation of a mini-grants program to disseminate evidence-based nutrition programs to rural churches and worksites. *Journal of Public Health Management and Practice*, *18*(5), 431-439. | US-Single State | 7 organizations | Cancer | Process | Documentation Review |
| Hager, E. R., Song, H. J., Lane, H. G., Guo, H. H., Jaspers, L. H., & Lopes, M. A. (2018). Pilot-testing an intervention to enhance wellness policy implementation in schools: Wellness Champions for Change. *Journal of nutrition education and behavior*, *50*(8), 765-775. | US-Single State | 6 school districts | School | Summative | Documentation Review |
| Heath, E., Sanon, V., Mast, D. K., Kibbe, D., & Lyn, R. (2021). Increasing community readiness for childhood obesity prevention: a case study of four communities in Georgia. *Health Promotion Practice*, *22*(5), 676-684. | US-Single State | 4 coalitions; community stakeholders in 2012 (n = 20) and 2017 (n = 18) | Child Welfare | Summative | Interview |
| Hefelfinger, J., Patty, A., Ussery, A., & Young, W. (2013). Technical assistance from state health departments for communities engaged in policy, systems, and environmental change: the ACHIEVE Program. | US-General | 64 individuals | Other | Summative | Documentation Review |
| Heisey‐Grove, D., & King, J. A. (2017). Physician and practice‐level drivers and disparities around meaningful use progress. *Health services research*, *52*(1), 244-267. | US-General | 865,370 physicians | EHR | Summative | Combination |
| House, L. D., Tevendale, H. D., & Martinez-Garcia, G. (2017). Implementing evidence-based teen pregnancy-prevention interventions in a community-wide initiative: building capacity and reaching youth. *Journal of Adolescent Health*, *60*(3), S18-S23. | US-General | 9 state and community-based organizations | Child Welfare | Combination | Documentation Review |
| Hunter, S. B., Chinman, M., Ebener, P., Imm, P., Wandersman, A., & Ryan, G. W. (2009). Technical assistance as a prevention capacity-building tool: a demonstration using the Getting to Outcomes® framework. *Health Education & Behavior*, *36*(5), 810-828. | US-General | 2 community coalitions | Substance Use | Process | Combination |
| Izquierdo, A., Ong, M., Pulido, E., Wells, K. B., Berkman, M., Linski, B., ... & Miranda, J. (2018). Community Partners in Care: 6-and 12-month outcomes of community engagement versus technical assistance to implement depression collaborative care among depressed older adults. *Ethnicity & Disease*, *28*(Suppl 2), 339. | US-Single State | 394 participants | Mental Health | Summative | Survey |
| Jadwin-Cakmak, L., Bauermeister, J. A., Cutler, J. M., Loveluck, J., Sirdenis, T. K., Fessler, K. B., ... & Harper, G. W. (2020). The health access initiative: A training and technical assistance program to improve health care for sexual and gender minority youth. *Journal of Adolescent Health*, *67*(1), 115-122. | US-Single State | 10 sites | Other | Summative | Interview |
| Jansen, A. L., Capesius, T. R., Lachter, R., Greenseid, L. O., & Keller, P. A. (2014). Facilitators of health systems change for tobacco dependence treatment: a qualitative study of stakeholders? perceptions. *BMC Health Services Research*, *14*(1), 1-10. | US-Single State | 18 key informants | Substance Use | Process | Combination |
| Johnson, L. E., Clará, W., Gambhir, M., Fuentes, R. C., Marín-Correa, C., Jara, J., ... & Azziz-Baumgartner, E. (2014). Improvements in pandemic preparedness in 8 Central American countries, 2008-2012. *BMC health services research*, *14*(1), 1-9. | Non-US- Multi Country | 8 Central American countries | Other | Summative | Documentation Review |
| Kahn, L., Hurth, J., Kasprzak, C. M., Diefendorf, M. J., Goode, S. E., & Ringwalt, S. S. (2009). The national early childhood technical assistance center model for long-term systems change. *Topics in Early Childhood Special Education*, *29*(1), 24-39. | US-General | 37 plans (summative), 32 plans (process) | Other | Summative | Combination |
| Kakietek, J., Dunn, L., O’Dell, S. A., Jernigan, J., & Khan, L. K. (2014). Peer Reviewed: Training and Technical Assistance for Compliance With Beverage and Physical Activity Components of New York City’s Regulations for Early Child Care Centers. *Preventing Chronic Disease*, *11*. | US-Single State | 174 group child care centers | Child Welfare | Summative | Documentation Review |
| Kauff, J. F., Clary, E., Lupfer, K. S., & Fischer, P. J. (2016). An evaluation of SOAR: implementation and outcomes of an effort to improve access to SSI and SSDI. *Psychiatric Services*, *67*(10), 1098-1102. | US-General | 92 individuals | Other | Summative | Combination |
| Kegeles, S. M., Rebchook, G., Pollack, L., Huebner, D., Tebbetts, S., Hamiga, J., ... & Zovod, B. (2012). An intervention to help community‐based organizations implement an evidence‐based HIV prevention intervention: The mpowerment project technology exchange system. *American Journal of Community Psychology*, *49*(1-2), 182-198. | US-General | 49 CBOs | HIV | Process | Combination |
| Kegler, M. C., & Redmon, P. B. (2006). Using technical assistance to strengthen tobacco control capacity: evaluation findings from the tobacco technical assistance consortium. *Public health reports*, *121*(5), 547-556. | US-General | 94 participants | Substance Use | Summative | Interview |
| Kennedy, C., Finkelstein, N., Hutchins, E., & Mahoney, J. (2004). Improving screening for alcohol use during pregnancy: the Massachusetts ASAP program. *Maternal and Child Health Journal*, *8*(3), 137-147. | US-Single State | 293 participants | Substance Use | Summative | Interview |
| Kershner, S., Flynn, S., Prince, M., Potter, S. C., Craft, L., & Alton, F. (2014). Using data to improve fidelity when implementing evidence-based programs. *Journal of Adolescent Health*, *54*(3), S29-S36. | US-Single State | 18 facilitators | Child Welfare | Process | Documentation Review |
| Kohler, F. W., Anthony, L. J., Steighner, S. A., & Hoyson, M. (2001). Teaching social interaction skills in the integrated preschool: An examination of naturalistic tactics. *Topics in Early Childhood Special Education*, *21*(2), 93-103. | Not reported | 4 preschoolders and 4 teachers; 39 students | School | Summative | Natural Observation |
| Kuehn, C., Tidwell, G., Vhugen, J., & Sharma, A. (2015). Lessons learned from transitioning PEPFAR Track 1.0 care and treatment programs: Case studies in financial management capacity building in Zambia and Botswana. *Journal of public health management and practice*, *21*(6), 564-572. | Non-US- Multi Country | 3 local partners | HIV | Summative | Documentation Review |
| Leake, R., Green, S., Marquez, C., Vanderburg, J., Guillaume, S., & Gardner, V. A. (2007). Evaluating the capacity of faith-based programs in Colorado. *Research on Social Work Practice*, *17*(2), 216-228. | US-Single State | 90 organizations total | Other | Summative | Interview |
| Lee, J. G., Ranney, L. M., Goldstein, A. O., McCullough, A., Fulton-Smith, S. M., & Collins, N. O. (2011). Successful implementation of a wellness and tobacco cessation curriculum in psychosocial rehabilitation clubhouses. *BMC Public Health*, *11*(1), 1-11. | US-Single State | 12 clubhouse staff | Substance Use | Summative | Interview |
| Leviton, L. C., Herrera, C., Pepper, S. K., Fishman, N., & Racine, D. P. (2006). Faith in action: Capacity and sustainability of volunteer organizations. *Evaluation and Program Planning*, *29*(2), 201-207. | US-General | 351 programs | Other | Summative | Survey |
| Li, Y., Spector, W. D., Glance, L. G., & Mukamel, D. B. (2012). State “technical assistance programs” for nursing home quality improvement: variations and potential implications. *Journal of aging & social policy*, *24*(4), 349-367. | US-General | Officials from 14 states | Other | Summative | Survey |
| Livet, M., Yannayon, M., Sheppard, K., Kocher, K., Upright, J., & McMillen, J. (2018). Exploring provider use of a digital implementation support system for school mental health: A pilot study. *Administration and Policy in Mental Health and Mental Health Services Research*, *45*(3), 362-380. | US-Region | 15 providers | Mental Health | Summative | Combination |
| Matsuoka, S., Obara, H., Nagai, M., Murakami, H., & Chan Lon, R. (2014). Performance-based financing with GAVI health system strengthening funding in rural Cambodia: a brief assessment of the impact. *Health Policy and Planning*, *29*(4), 456-465. | Non-US-Multi-Region | 10 operational districts | Other | Summative | Not Reported |
| Mayberry, R. M., Daniels, P., Yancey, E. M., Akintobi, T. H., Berry, J., Clark, N., & Dawaghreh, A. (2009). Enhancing community-based organizations’ capacity for HIV/AIDS education and prevention. *Evaluation and program planning*, *32*(3), 213-220. | US-General | 24 participants in year 1, 23 participants in year 2, 22 participants in year 3 | HIV | Summative | Survey |
| McInerney, M., & Hamilton, J. L. (2007). Elementary and middle schools technical assistance center: An approach to support the effective implementation of scientifically based practices in special education. *Exceptional Children*, *73*(2), 242-255. | US-General | 32 local school districts | School | Summative | Combination |
| Mehta, P., Brown, A., Chung, B., Jones, F., Tang, L., Gilmore, J., ... & Wells, K. (2017). Community Partners in Care: 6-month outcomes of two quality improvement depression care interventions in male participants. *Ethnicity & Disease*, *27*(3), 223. | US-Single State | 60 agencies, 95 programs; 423 males | Mental Health | Summative | Survey |
| Merlo, C. L., Michael, S., Brener, N. D., & Blanck, H. (2018). Peer Reviewed: State-Level Guidance and District-Level Policies and Practices for Food Marketing in US School Districts. *Preventing Chronic Disease*, *15*. | US-General | state-level Nutrition Services (n = 51) district-level Nutrition Services questionnaire (n = 660 districts) district-level General School Environment questionnaire (n = 630 districts) | School | Summative | Survey |
| Meyerson, B. E., Barnes, P. R., King, J., Degi, L. S., Halverson, P. K., & Polmanski, H. F. (2015). Measuring accreditation activity and progress: findings from a survey of Indiana local health departments, 2013. *Public Health Reports*, *130*(5), 447-452. | US-Single State | 71 participants | Other | Summative | Survey |
| Mitchell, R. E., Stone-Wiggins, B., Stevenson, J. F., & Florin, P. (2004). Cultivating capacity: Outcomes of a statewide support system for prevention coalitions. *Journal of prevention & intervention in the community*, *27*(2), 67-87. | US-Single State | 41 health-oriented coalitions | Other | Summative | Interview |
| Mokotoff, E. D., Ruth, K. G., Benbow, N., Sweeney, P., Sapiano, T. N., & McNaghten, A. D. (2019). Data to care: lessons learned from delivering technical assistance to 20 health departments. *JAIDS Journal of Acquired Immune Deficiency Syndromes*, *82*, S74-S79. | US-General | 20 states | HIV | Combination | Combination |
| Moreland-Russell, S., Adsul, P., Nasir, S., Fernandez, M. E., Walker, T. J., Brandt, H. M., ... & Brownson, R. C. (2018). Evaluating centralized technical assistance as an implementation strategy to improve cancer prevention and control. *Cancer Causes & Control*, *29*(12), 1221-1230. | US-General | 39 participants | Cancer | Summative | Survey |
| Morton, I., Hurley, B., Castillo, E. G., Tang, L., Gilmore, J., Jones, F., ... & Wells, K. (2020). Outcomes of two quality improvement implementation interventions for depression services in adults with substance use problems. *The American Journal of Drug and Alcohol Abuse*, *46*(2), 251-261. | US-Single State | 95 programs and 588 participants | Mental Health | Summative | Survey |
| Ngo, V. K., Sherbourne, C., Chung, B., Tang, L., Wright, A. L., Whittington, Y., ... & Miranda, J. (2016). Community engagement compared with technical assistance to disseminate depression care among low-income, minority women: A randomized controlled effectiveness study. *American Journal of Public Health*, *106*(10), 1833-1841. | US- City | 595 female individuals | Mental Health | Summative | Survey |
| O'Donnell, L., Scattergood, P., Adler, M., Doval, A. S., Barker, M., Kelly, J. A., Kegeles, S. M., Rebchook, G. M., Adams, J., Terry, M. A., & Neumann, M. S. (2000). The role of technical assistance in the replication of effective HIV interventions. AIDS education and prevention : official publication of the International Society for AIDS Education, 12(5 Suppl), 99–111. | US-Single State | 28 personnel | HIV | Process | Combination |
| Oliva, G., Rienks, J., & Chavez, G. F. (2007). Evaluating a program to build data capacity for core public health functions in local maternal child and adolescent health programs in California. *Maternal and child health journal*, *11*(1), 1-10. | US-Single State | 61 MCH directors | Other | Summative | Combination |
| Olson, J. R., McCarthy, K. J., Perkins, D. F., & Borden, L. M. (2018). A formative evaluation of a coach-based technical assistance model for youth-and family-focused programming. *Evaluation and Program Planning*, *67*, 29-37. | US-General | 31 individuals in 2015; 28 individuals in 2016 | Other | Formative | Survey |
| Olson, J. R., Coldiron, J. S., Parigoris, R. M., Zabel, M. D., Matarese, M., & Bruns, E. J. (2020). Developing an evidence-based technical assistance model: A process evaluation of the National Training and Technical Assistance Center for Child, Youth, and Family Mental Health. *The Journal of Behavioral Health Services & Research*, *47*(3), 312-330. | US-General | 102 grantees | Mental Health | Combination | Combination |
| Ong, M. K., Jones, L., Aoki, W., Belin, T. R., Bromley, E., Chung, B., ... & Wells, K. (2017). A community-partnered, participatory, cluster-randomized study of depression care quality improvement: three-year outcomes. *Psychiatric services*, *68*(12), 1262-1270. | US- City | 600 individuals from 89 community agency programs | Mental Health | Summative | Documentation Review |
| Osborne, C., Bobbitt, K. C., Boelter, J. M., & Ayrhart, T. K. (2014). Early lessons learned from building local early childhood comprehensive systems in Texas. *Journal of Applied Research on Children: Informing Policy for Children at Risk*, *5*(1), 7. | US-Single State | 7 communities | Child Welfare | Formative | Combination |
| Palinkas, L. A., Campbell, M., & Saldana, L. (2018). Agency leaders' assessments of feasibility and desirability of implementation of evidence-based practices in youth-serving organizations using the stages of implementation completion. *Frontiers in Public Health*, *6*, 161. | US-Single State | 19 agency chief executive officers and program directors | Other | Summative | Interview |
| Parks, C. A., Stern, K. L., Fricke, H. E., Clausen, W., & Yaroch, A. L. (2020). Healthy food incentive programs: findings from food insecurity nutrition incentive programs across the United States. *Health promotion practice*, *21*(3), 421-429. | US-General | 30 interviews with 38 people: 22 interviews across 19 grantee organizations were conducted | Other | Summative | Interview |
| Reibstein, R. (2008). Does providing technical assistance for toxics use reduction really work? A program evaluation utilizing toxics use reduction act data to measure pollution prevention performance. *Journal of Cleaner Production*, *16*(14), 1494-1506. | US-General | 612 in nonvisit group and 443 facilities in TA group | Other | Summative | Documentation Review |
| Rogers, S. J., Ahmed, M., Hamdallah, M., & Little, S. (2010). Garnering grantee buy-in on a national cross-site evaluation: The case of ConnectHIV. *American Journal of Evaluation*, *31*(4), 447-462. | US-General | Individuals from 15 of the 20 grantees | HIV | Summative | Survey |
| Rosselli, R. T., Davis, M. K., Simeonsson, K., Johnson, M., Goode, B., Casani, J., & MacDonald, P. D. (2010). An academic/government partnership to provide technical assistance with pandemic influenza planning to local health departments in North Carolina. *Public Health Reports*, *125*(5_suppl), 92-99. | US-Single State | 79 LHDs | Other | Combination | Combination |
| Ruprah, I., & Marcano, L. (2009). Does technical assistance matter? an impact evaluation approach to estimate its value added. *Journal of Development Effectiveness*, *1*(4), 507-528. | Non-US- Multi Country | 511 participants | Other | Summative | Documentation Review |
| Rushovich, B. R., Bartley, L. H., Steward, R. K., & Bright, C. L. (2015). Technical assistance: a comparison between providers and recipients. *Human Service Organizations: Management, Leadership & Governance*, *39*(4), 362-379. | US-General | 47 participants | Child Welfare | Process | Combination |
| Ruzek, J. I., Landes, S. J., McGee-Vincent, P., Rosen, C. S., Crowley, J., Calhoun, P. S., ... & Kirchner, J. E. (2020). Creating a practice-based implementation network: Facilitating practice change across health care systems. *The Journal of Behavioral Health Services & Research*, *47*(4), 449-463. | US-General | 18 VA clinics | Mental Health | Summative | Combination |
| Ryan, A. M., Bishop, T. F., Shih, S., & Casalino, L. P. (2013). Small physician practices in New York needed sustained help to realize gains in quality from use of electronic health records. *Health Affairs*, *32*(1), 53-62. | US-Single State | 360 physicians | EHR | Summative | Documentation Review |
| Ryan, A. M., McCullough, C. M., Shih, S. C., Wang, J. J., Ryan, M. S., & Casalino, L. P. (2014). The intended and unintended consequences of quality improvement interventions for small practices in a community-based electronic health record implementation project. *Medical care*, 826-832. | US-Single State | 143 healthcare practices | EHR | Summative | Documentation Review |
| Satterlund, T. D., Treiber, J., Kipke, R., Kwon, N., & Cassady, D. (2013). Accommodating diverse clients’ needs in evaluation capacity building: A case study of the Tobacco Control Evaluation Center. *Evaluation and program planning*, *36*(1), 49-55. | US-Single State | Not reported | Substance Use | Summative | Combination |
| Segre, L. S., O’Hara, M. W., & Fisher, S. D. (2013). Perinatal depression screening in Healthy Start: an evaluation of the acceptability of technical assistance consultation. *Community mental health journal*, *49*(4), 407-411. | US-General | 11 programs | Other | Summative | Combination |
| Sharpe, P. A., Flint, S., Burroughs-Girardi, E. L., Pekuri, L., Wilcox, S., & Forthofer, M. (2015). Building capacity in disadvantaged communities: development of the community advocacy and leadership program. *Progress in community health partnerships: research, education, and action*, *9*(1), 113. | US-Single State | 3 community groups and 6 group participants | Other | Process | Combination |
| Sharpe, P. A., Wilcox, S., Stucker, J., Kinnard, D., Bernhart, J., & James, K. L. (2020). Community health advisors' characteristics and behaviors, role performance, and volunteer satisfaction in a church-based healthy eating and physical activity intervention. *Journal of community health*, *45*(1), 88-97. | US-Single State | 61 pastors and 71 program coordinators | Other | Combination | Survey |
| Sherbourne, C. D., Aoki, W., Belin, T. R., Bromley, E., Chung, B., Dixon, E., ... & Wells, K. (2017). Comparative effectiveness of two models of depression services quality improvement in health and community sectors. *Psychiatric services*, *68*(12), 1315-1320. | US- City | 1018 individuals | Mental Health | Summative | Survey |
| Solomon, G. T., Bryant, A., May, K., & Perry, V. (2013). Survival of the fittest: Technical assistance, survival and growth of small businesses and implications for public policy. *Technovation*, *33*(8-9), 292-301. | US-General | 19,533 business records | Other | Summative | Survey |
| Solomon, G., & Perry, V. G. (2011). Looking out for the little guy: The effects of technical assistance on small business financial performance. *Journal of Marketing Development and Competitiveness*, *5*(4), 21-31. | US-General | 447 business respondents | Other | Summative | Survey |
| Spadaro, A. J., Grunbaum, J. A., Dawkins, N. U., Wright, D. S., Rubel, S. K., Green, D. C., & Simoes, E. J. (2011) Training and Technical Assistance to Enhance Capacity Building Between Prevention Research Centers and Their Partners. *Prev Chronic Disease, 8*(3). | US-General | 9 prevention research centers included in sample for interviews | Other | Process | Interview |
| Spoth, R., Clair, S., Greenberg, M., Redmond, C., & Shin, C. (2007). Toward dissemination of evidence-based family interventions: maintenance of community-based partnership recruitment results and associated factors. *Journal of Family Psychology*, *21*(2), 137. | US- Multi State | 2,650 participants | Substance Use | Summative | Combination |
| Springgate, B., Tang, L., Ong, M., Aoki, W., Chung, B., Dixon, E., ... & Wells, K. B. (2018). Comparative effectiveness of coalitions versus technical assistance for depression quality improvement in persons with multiple chronic conditions. *Ethnicity & Disease*, *28*(Suppl 2), 325. | US-Single State | 93 programs from 50 agencies, 548 participants | Mental Health | Summative | Survey |
| Stevenson, J. F., Florin, P., Mills, D. S., & Andrade, M. (2002). Building evaluation capacity in human service organizations: A case study. *Evaluation and Program Planning*, *25*(3), 233-243. | US-Single State | 25 different participants from the 13 agencies. | Substance Use | Summative | Combination |
| Strunk, K. O., McEachin, A., & Westover, T. N. (2014). The Use and Efficacy of Capacity‐Building Assistance for Low‐Performing Districts: The Case of California's District Assistance and Intervention Teams. *Journal of Policy Analysis and Management*, *33*(3), 719-751. | US-Single State | 95 districts total involving 20.3 million students | School | Combination | Survey |
| Sugarman, J. R., Phillips, K. E., Wagner, E. H., Coleman, K., & Abrams, M. K. (2014). The safety net medical home initiative: transforming care for vulnerable populations. *Medical care*, S1-S10. | US-General | 65 safety net practices in 5 states | Other | Combination | Survey |
| Tang, K. C., Nutbeam, D., Kong, L., Wang, R., & Yan, J. (2005). Building capacity for health promotion—a case study from China. *Health promotion international*, *20*(3), 285-295. | Non-US-Multi-City | 30 practitioners | Other | Combination | Survey |
| Todo, Y. (2011). Impacts of aid-funded technical assistance programs: Firm-level evidence from the Indonesian foundry industry. *World Development*, *39*(3), 351-362. | Non-US-Country | 150 foundry firms | Other | Summative | Survey |
| Treiber, J., Cassady, D., Kipke, R., Kwon, N., & Satterlund, T. (2011). Building the evaluation capacity of California’s local tobacco control programs. *Health promotion practice*, *12*(6_suppl_2), 118S-124S. | US-Single State | 79 projects involving responses from 103 directors | Substance Use | Combination | Combination |
| Valdivia, M. (2015). Business training plus for female entrepreneurship? Short and medium-term experimental evidence from Peru. *Journal of Development Economics*, *113*, 33-51. | Non-US-Multi-City | 1979 individuals | Other | Summative | Combination |
| Vogel, W. M., Noether, C. D., & Steadman, H. J. (2007). Preparing communities for re-entry of offenders with mental illness: The ACTION approach. *Journal of Offender Rehabilitation*, *45*(1-2), 167-188. | US- Multi City | 8 sites from outcome evaluation, but only 3 sites received TA | Other | Summative | Combination |
| Walkinshaw, L. P., Mason, C., Allen, C. L., Vu, T., Nandi, P., Santiago, P. M., & Hannon, P. A. (2015). Process evaluation of a regional public health model to reduce chronic disease through policy and systems changes, Washington State, 2010–2014. | US-Single State | 14 WA DOH leadership and staff members, 11 LHJ leadership and staff members, 4 external consultants, and 5 other regional partners | Other | Process | Combination |
| Ward, D. S., Vaughn, A. E., Mazzucca, S., & Burney, R. (2017). Translating a child care based intervention for online delivery: development and randomized pilot study of Go NAPSACC. *BMC Public Health*, *17*(1), 1-14. | US-Single State | 33 centers, which were randomized to immediate (intervention, n = 18) or delayed (control, n = 15) | Child Welfare | Process | Combination |
| Watson, D. P., Ahonen, E. Q., Shuman, V., Brown, M., Tsemberis, S., Huynh, P., ... & Xu, H. (2018). The housing first technical assistance and training (HFTAT) implementation strategy: outcomes from a mixed methods study of three programs. *Substance abuse treatment, prevention, and policy*, *13*(1), 1-13. | US-General | 113 participants | Housing | Combination | Combination |
| Watson-Thompson, J., Woods, N. K., Schober, D. J., & Schultz, J. A. (2013). Enhancing the capacity of substance abuse prevention coalitions through training and technical assistance. *Journal of prevention & intervention in the community*, *41*(3), 176-187. | US-Region | 7 coalitions | Substance Use | Summative | Survey |
| Watson‐Thompson, J., Woods, N. K., Schober, D. J., & Schultz, J. A. (2014). Implementing the capacity building for change model with substance abuse prevention coalitions. *Journal of Community Psychology*, *42*(6), 748-763. | US-Region | 8 coalitions (27 individuals) | Substance Use | Summative | Combination |
| Weinberg, L. A., Zetlin, A., & Shea, N. M. (2009). Removing barriers to educating children in foster care through interagency collaboration: a seven county multiple-case study. *Child welfare*, *88*(4). | US-Single State | 7 counties | Child Welfare | Combination | Combination |
| Yazejian, N., & Iruka, I. U. (2015). Associations among tiered quality rating and improvement system supports and quality improvement. *Early Childhood Research Quarterly*, *30*, 255-265. | US-Single State | 412 sites implementing TQRIS | Child Welfare | Summative | Survey |
| Young-Pelton, C. A., & Doty, D. (2013). Improving educational programs for students with autism in rural schools: A preliminary program description of the Montana Autism Education Project. *Rural Special Education Quarterly*, *32*(3), 24-32. | US-Single State | 100 schools received onsite TA visits | School | Process | Documentation Review |
| Young, B. R., Leeks, K. D., Bish, C. L., Mihas, P., Marcelin, R. A., Kline, J., & Ulin, B. F. (2020). Community-University Partnership Characteristics for Translation: Evidence From CDC's Prevention Research Centers. *Frontiers in Public Health*, *8*, 79. | US-General | 14 stakeholders (initiators N = 10; adopters N = 4). | Other | Summative | Interview |
| Youngblood, D. J., Dvorak, B. I., Woldt, W. E., Hawkey, S. A., & Hygnstrom, J. R. (2008). Quantifying and comparing a P2 program's benefits: pollution prevention technical assistance in Nebraska. *Journal of Cleaner Production*, *16*(6), 761-770. | US-Single State | 75 clients | Other | Summative | Combination |
